# BLIT: an R package for seamless integration of command-line bioinformatics tool universe

**DOI:** 10.1093/bioadv/vbag088

**Published:** 2026-03-24

**Authors:** Jia Ding, Yun Peng, Ruochen Wei, Boquan Wang, Jian-Guo Zhou, Shixiang Wang

**Affiliations:** Department of Biomedical Informatics, School of Life Sciences, Central South University, Changsha, 410013, P. R. China; Department of Urology, Peking University People’s Hospital, Beijing, 100044, P. R. China; Department of Biomedical Informatics, School of Life Sciences, Central South University, Changsha, 410013, P. R. China; Department of Biomedical Informatics, School of Life Sciences, Central South University, Changsha, 410013, P. R. China; Department of Oncology, The Second Affiliated Hospital of Zunyi Medical University, Zunyi, 563000, P. R. China; Department of Biomedical Informatics, School of Life Sciences, Central South University, Changsha, 410013, P. R. China

## Abstract

**Motivation:**

Modern computational biology relies heavily on command-line tools that underpin analytical workflows, yet integrating these tools into R-based analyses poses substantial challenges for reproducibility, especially for researchers with limited computational expertise. While R excels at statistical analysis and visualization, its native capabilities for executing external commands remain rudimentary, lacking structured integration, environment management, and reliable cross-platform support.

**Results:**

To address these limitations, BLIT, an R package, provides a unified framework for seamless command-line integration within R, enabling R users to invoke external tools in an intuitive manner. Rather than positioning itself as yet another workflow manager, BLIT emphasizes the robust encapsulation and coordination of command-line tools, enabling users to construct reproducible analysis pipelines without leaving the R environment. BLIT encapsulates command-line programs as R6 objects with dynamic validation, Micromamba-based environment management, and native piping support. This makes BLIT a direct bridge between R scripts and heterogeneous command-line ecosystems, replacing fragile system() calls with a robust, standardized framework that supports conditional execution and single-machine parallel computing, with the capability to hand off constructed command workflows to HPC schedulers or workflow engines for large-scale execution. Although designed for bioinformatics, BLIT offers a general, platform-agnostic solution for constructing reproducible analytical pipelines within R.

**Availability and implementation:**

BLIT is available on CRAN (https://cran.r-project.org/package=blit) and on GitHub (https://github.com/WangLabCSU/blit).

## 1 Introduction

The field of biomedical research is undergoing a paradigm shift driven by the explosive growth of data generated by high-throughput sequencing, multi-omics profiling, and advanced single-cell technologies (Liu *et al.* 2025). These features present significant computational challenges. Navigating such complexity often involves labor-intensive operations: switching between specialized tools, coordinating data formats, and managing fragmented workflows. This manual process is time-consuming, error-prone, and limits reproducibility.

Within this context, R has become central for statistical analysis and visualization in biomedical data science. However, R exhibits key limitations: native mechanisms for invoking system commands and external programs remain cumbersome, and its pathway for handling biomedical big data is often less straightforward, particularly in scenarios requiring cross-tool coordination and potential scaling to large computational workloads.

These challenges are amplified within a fragmented computational landscape. Bioinformatics workflows typically rely on a combination of technologies, each with distinct strengths but creating an interoperability gap: command-line tools (including widely used Python and Perl scripts) excel at batch processing and stream manipulation but are challenging for non-specialists to use, particularly when attempting to integrate them into R-based workflows due to complex parameter syntax, the need to understand redirection logic, and difficulties debugging opaque error messages; R provides powerful statistical analysis and visualization capabilities but offers relatively limited native support for integrating external tools, often requiring additional scripting when coordinating large-scale external computations. This fragmentation forces researchers to constantly switch environments, reformat data, and debug complex pipelines (Williams *et al.* 2024). More critically, this ecosystem fragmentation contributes to profound methodological inconsistencies and a reproducibility crisis, as was revealed through inter-laboratory comparisons in microbiome analysis by O’Sullivan et al. (2021). These limitations are not simply workflow-level inconveniences but stem fundamentally from the difficulty of integrating heterogeneous command-line tools into R in a reproducible and structured way.

To address these issues, unified frameworks are imperative to combine technological strengths while hiding underlying complexity. While existing R packages like processx (Csárdi and Chang 2025) provide low-level process execution, they often lack higher-level abstractions for structured command construction and workflow composition. Motivated by this need, we developed BLIT, an R package that seamlessly integrates command-line tools (including Python and Perl scripts) into R workflows. It is designed to address the challenges R users face in organizing and managing external commands and to provide a convenient path for single-machine parallel computing within R, with the ability to hand off constructed command workflows to dedicated workflow managers or HPC schedulers for large-scale execution. By abstracting commands into composable objects and supporting the direct conversion of native R data structures into command-line inputs, BLIT significantly reduces system integration complexity and provides a novel solution for building robust, scalable bioinformatics pipelines. Specifically, BLIT extends R’s command-line interface capabilities, providing a command-centric abstraction that can also serve as a foundational building block for more complex workflows when needed.

## 2 Tool description

### 2.1 Design philosophy and overview

**Figure 1:**
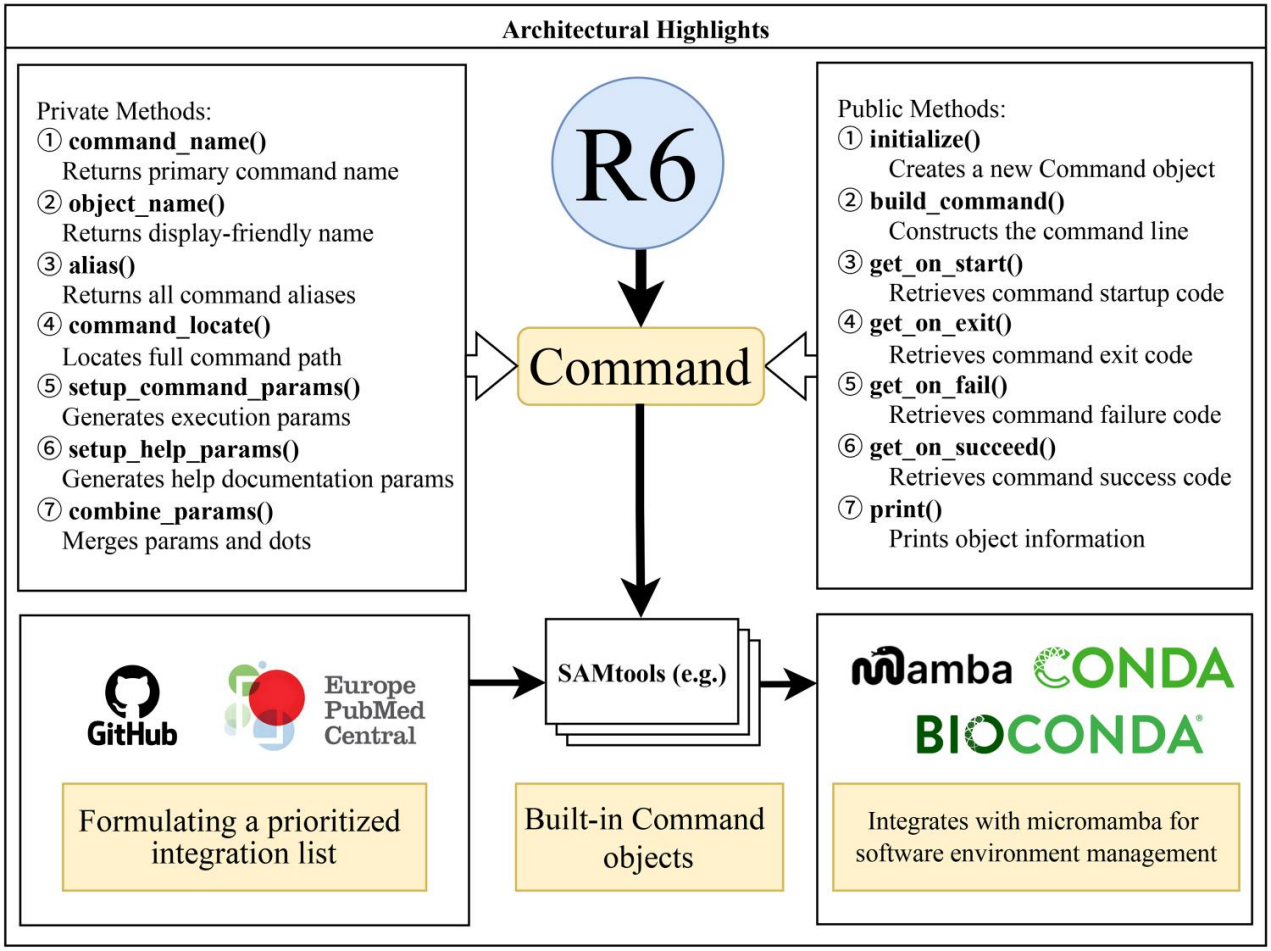
BLIT design architecture.

The development of BLIT follows a clear technical trajectory (Fig. 1): it begins with the implementation of a core R6-based command engine for shell command translation and pipeline chaining. Building upon this foundation, the project employs bibliometric analysis to identify and prioritize high-impact bioinformatics tools. Finally, prebuilt wrappers are developed for these prioritized tools [e.g. SAMtools (Li *et al.* 2009)], integrated with the lightweight Micromamba environment for seamless support of the Conda/Bioconda (Grüning *et al.* 2018) ecosystem. This process ensures cross-platform reproducibility and improves the readability and maintainability of analytical workflows.

### 2.2 Tool prioritization via bibliometric screening and community signals

To ensure BLIT addresses critical bioinformatics needs, we employed a structured, dual-perspective approach for prioritizing command-line tools for wrapping. We established a data-driven tool prioritization strategy through a two-phase process. First, we compiled an initial candidate dictionary of approximately 60 command-line bioinformatics tools (Table 1, available as supplementary data at *Bioinformatics Advances* online). Subsequently, we evaluated these tools from dual perspectives: academic impact and community adoption. Academic impact was quantified by analyzing publication frequency (2022–24) in the Europe PMC database using a custom R script (Table 2, available as supplementary data at *Bioinformatics Advances* online), with search queries combining tool names and qualifiers like “bioinformatics” to minimize ambiguity. Community adoption was assessed via GitHub stars and Bioconda download trends. By synthesizing these metrics, we generated a prioritized list (Table 3, available as supplementary data at *Bioinformatics Advances* online) organized into Genomics, Transcriptomics, and General tools, detailing usage frequency, category, and descriptions. This workflow ensures BLIT’s development aligns with both academic relevance and practical utility.

### 2.3 An object-oriented framework for command composition and execution

**Figure 2:**
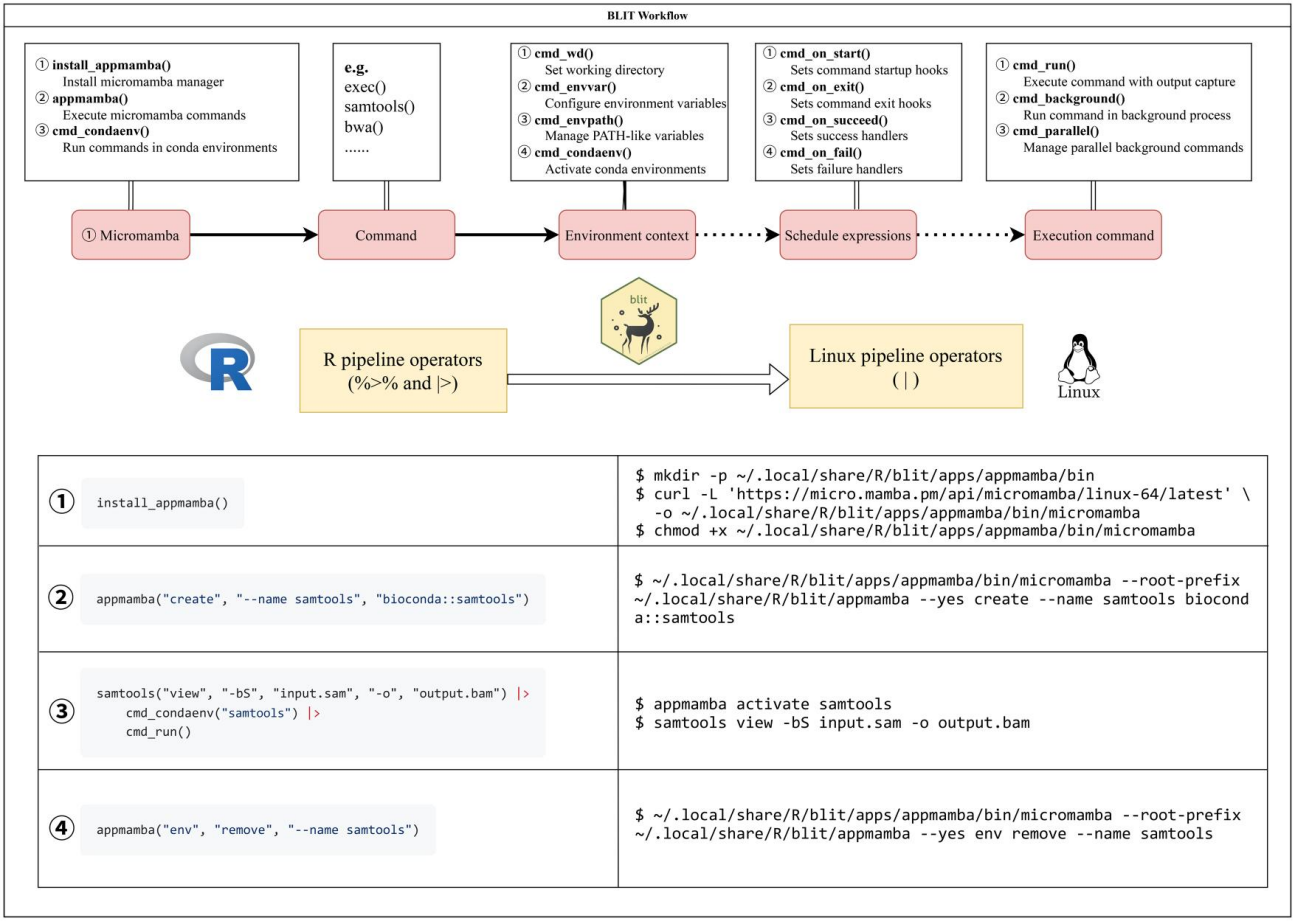
Workflow of the BLIT package.

At the heart of BLIT lies an object-oriented framework designed to bridge the gap between R and shell environments. As illustrated in Fig. 2, the complete workflow begins with environment configuration and proceeds through command construction to flexible execution. The framework reinterprets native R pipeline operators (%>% and |>) as Unix-style pipes (|), allowing users to construct intuitive, shell-like workflows directly within R. This automatic translation preserves the readability and chaining syntax of tidyverse-style R code while enabling transparent interoperation with complex command-line processes.

The core of BLIT’s command-line interaction is an R6-based object-oriented framework. Its core function, exec(), encapsulates a command-line tool and its arguments into a structured R object, rather than a traditional string. For instance, exec(“samtools,” “view,” “-bS,” “input.sam”) creates an executable command object. This paradigm shift yields a fundamental advantage: commands become first-class citizens in the R environment that can be safely manipulated, composed, and passed around.

Specialized wrapper functions (e.g. samtools()) built upon exec() provide simplified interfaces for common tools. These functions inherit the core object model, enhancing usability while preserving the flexibility and transparency of the underlying commands.

Command execution builds upon this object model. cmd_run() handles sequential execution, while cmd_parallel() leverages parallel backends such as the future framework for concurrency. Crucially, because commands are objects, complex composition patterns become a natural extension of the design. Users can combine multiple commands into a sequential sub-pipeline using R’s native pipe operator (|>), then process multiple instances of this sub-pipeline object concurrently. This pattern implements a nested execution pattern, coordinating sample-level parallelism with internal sequential steps. A unified execution interface ensures consistent behavior across modes.

Note on Scalability: The cmd_parallel() function is designed to provide a convenient interface for parallel execution on a single multi-core machine, forming a user-friendly command-orchestration layer. It is important to note that BLIT does not include native integration with cluster schedulers or job prioritization. For large-scale workloads requiring scheduling across nodes, command sequences constructed with BLIT can be handed off to dedicated workflow managers or HPC schedulers. Users should also be mindful of resource constraints: potential CPU oversubscription (when commands are themselves multi-threaded), I/O contention from many concurrent processes, and operating system limits on processes or file descriptors. These can be managed by configuring the global worker count and per-command thread parameters appropriately.

For example, the following code demonstrates how to execute an internal serial analysis pipeline (QC, alignment, sorting) in parallel for multiple samples:


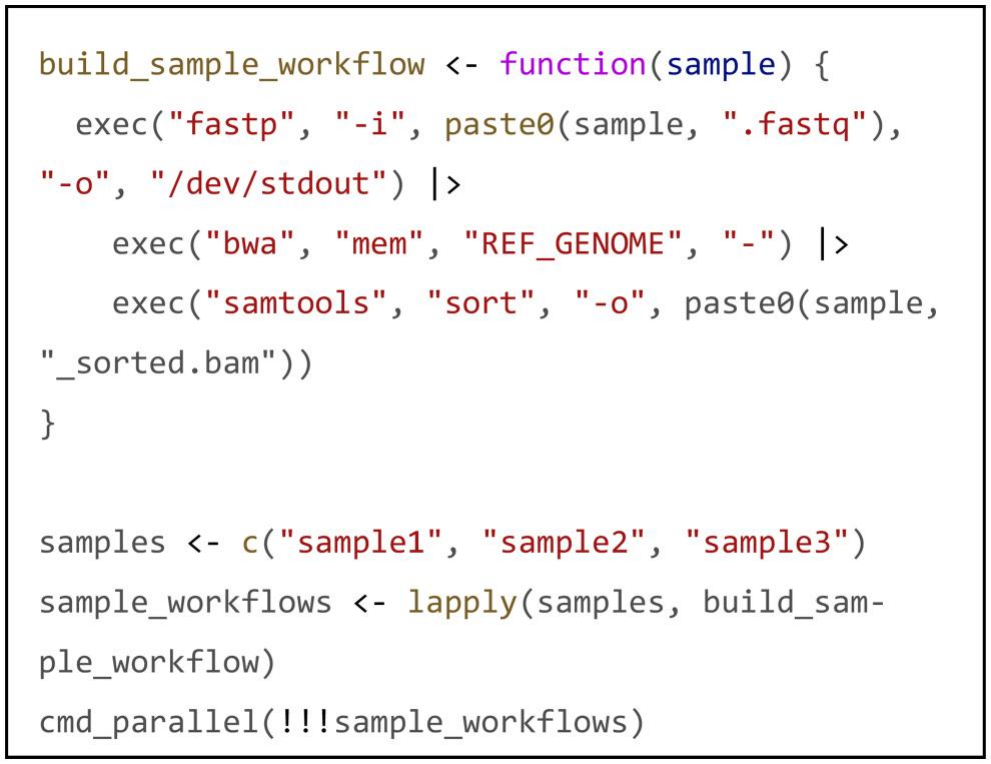


Unified interfaces ensure predictable behavior across execution modes, minimizing errors and easing transitions between exploration and production. This enables developers to construct complex, reproducible pipelines in a concise and intuitive manner.

### 2.4 Modular environment management and declarative workflow control

BLIT ensures computational reproducibility through robust environment management, built on the lightweight Micromamba engine—the statically linked, self-contained executable version of Mamba. This foundation enables fast dependency resolution with minimal overhead and seamless installation and execution of tools from the Conda/Bioconda ecosystems across platforms.

Building on this environment foundation, BLIT provides modular environment configuration utilities. The cmd_wd() function allows specification of working directories for scoped command execution, while cmd_envvar() and cmd_envpath() manage custom environment variables and dynamic path resolution. These abstractions ensure reliable invocation of external binaries across user-defined directory trees and shared computing environments.

Core Micromamba operations are directly accessible. The install_appmamba() function deploys Micromamba binaries, cmd_condaenv() dynamically configures environment path variables within Conda environments, and appmamba() executes environment operations such as creation. After initializing environments via appmamba(), users can seamlessly execute bioinformatics tools within these managed environments using BLIT’s workflow functions.

Workflow logic is enhanced by schedule expressions. Lifecycle callbacks are supported through hooks like cmd_on_start() and cmd_on_exit() for pre/post tasks, and cmd_on_succeed() or cmd_on_fail() for conditional branching, fault tolerance, and error reporting. This mechanism enables the design of reproducible, fault-resilient workflows that automatically handle edge cases.

## 3 Comparison

### 3.1 The advantage of exec() over system()

R’s native system() and system2() functions require users to construct commands as monolithic strings, a paradigm that becomes critically fragile in complex, real-world scenarios. Consider a data processing workflow combining piping and file redirection: compressing a file, piping its stream to another process for decompression, and redirecting the output to a new file. Implementing this with system() necessitates crafting an error-prone string with correct escaping for all special characters:


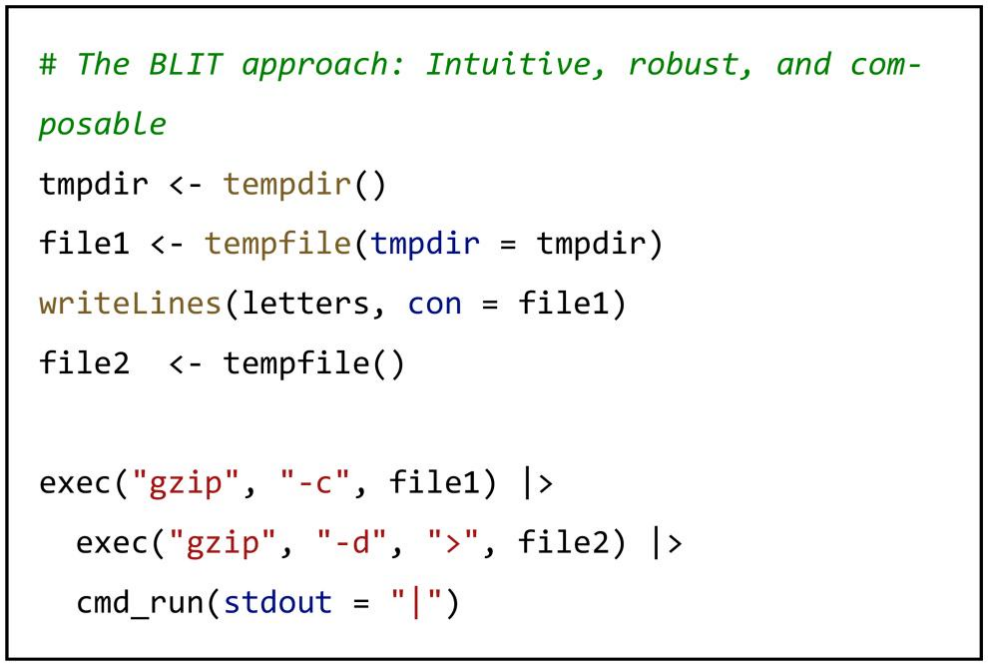


While packages like glue (Hester and Bryan 2024) provide readable string interpolation, BLIT advances the paradigm further: its exec() function abstracts commands into structured, composable R objects. The same complex operation is represented as a series of intuitive, composable elements, with BLIT automatically and reliably handling the underlying piping and redirection logic.

The core contribution is that exec() establishes an extensible, object-oriented abstraction layer. This layer serves as the common foundation enabling BLIT’s three integrated capabilities:

- Native Integration with R Operators: Command objects can be directly composed using R’s pipe (|>) and redirection (>) operators, with BLIT automatically handling the underlying shell semantics translation.
- A Vehicle for Standardized Logging and Hooks: The object lifecycle provides a unified attachment point for execution hooks like cmd_on_start() and cmd_on_exit().
- A Foundation for Seamless Environment Management: Command objects can be dynamically associated with BLIT’s environment management features, ensuring execution within the correct dependency context.

Thus, exec() transforms command-line interaction from ad-hoc string manipulation into an engineering practice based on predictable, composable, and decoratable objects, providing a unified foundation for reliable CLI integration within R.

### 3.2 Positioning within the workflow management ecosystem

BLIT’s design addresses a specific need within the existing tool ecosystem. It is not a competitor to workflow managers like targets (Landau 2021), Nextflow (Di Tommaso et al. 2017), or Snakemake (Köster and Rahmann 2012) but plays a complementary role. These powerful workflow managers excel at task-level orchestration—managing complex dependencies, job scheduling, and computational resource scaling for large workloads. For R users, however, the initial phase of constructing and validating command-line steps often occurs interactively within environments like RStudio. BLIT is designed for this phase: it transforms command-line tools into structured, callable R objects, enabling users to build and test execution logic using native R syntax and interactive debugging. This makes BLIT a command-orchestration layer with developer efficiency as its advantage, ideal for interactive prototyping and lightweight analysis within the R environment.


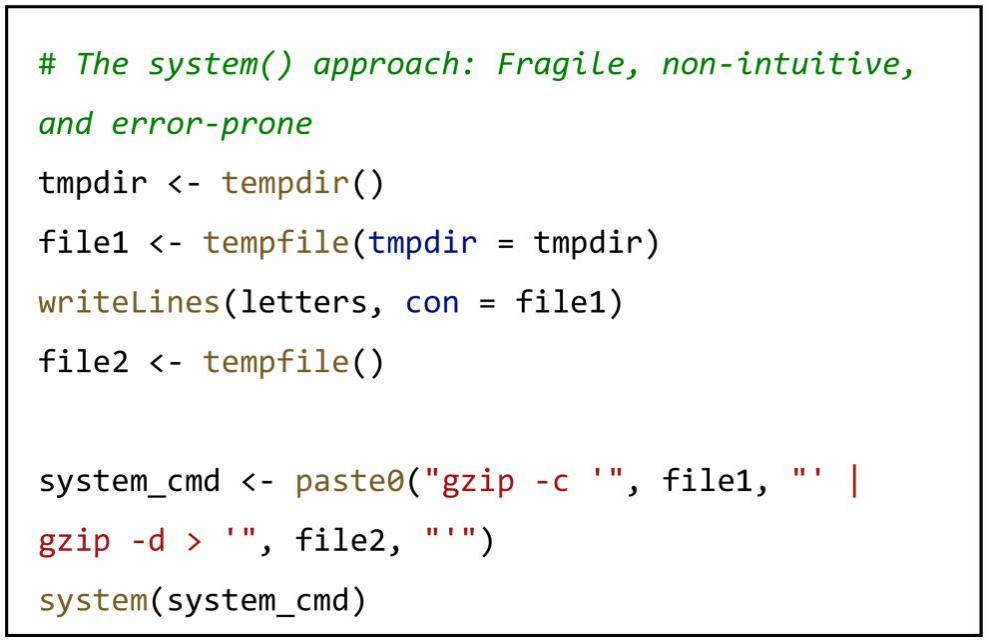


Critically, this R-native approach significantly lowers the barrier to entry. Users proficient in R and basic shell commands can immediately productively use BLIT, without first learning the additional specialized syntax of workflow tools. It is important to reiterate that BLIT’s concurrency model is designed for single-machine parallelism, in contrast to the distributed, multi-node scaling capabilities of full-featured workflow managers. Furthermore, the command logic developed and validated in BLIT can be exported as standalone scripts, which can then be integrated as R script within larger Snakemake or Nextflow pipelines, thereby combining the agility of BLIT for prototyping with the robustness of established managers for production scaling.

It should be noted that while BLIT excels in command-level integration and interactive use, we acknowledge certain limitations compared to established workflow managers. Specifically, checkpoint and resume capabilities remain under active development in BLIT. These features are particularly important for managing workflows with extended execution times. Meanwhile, we recognize that established platforms like Snakemake and Nextflow benefit from robust and extensive plugin and community ecosystems. As a newer tool, BLIT’s ecosystem is still in its early stages of growth. Consequently, fostering BLIT’s own community and extension ecosystem is a key priority on its future development roadmap.

A detailed comparative table is available in Table 4, available as supplementary data at *Bioinformatics Advances* online), analyzing various dimensions systematically to clarify their distinct design philosophies and suitable application scenarios.

## 4 Implementation

### 4.1 Systematic testing and performance optimization

BLIT’s robustness was validated through unit and integration testing of core R6 components, as well as empirical evaluation on clinical RNA-seq datasets. Redundant attributes in command objects were removed to reduce memory usage and improve execution speed. Continuous integration on GitHub Actions ensures cross-platform consistency, while parallelization and caching mitigate performance bottlenecks. These measures collectively ensure that BLIT is a reliable and user-friendly system, adaptable to diverse bioinformatics workflows.

To further validate BLIT’s completeness and engineering value in real-world complex scenarios, we performed a complete, production-ready functional re-implementation and engineering encapsulation of an open-source, modular, and community-utilized medium-complexity circRNA analysis pipeline using BLIT. This case study (original: https://github.com/OncoHarmony-Network/circrna-pipeline; BLIT version: https://github.com/WangLabCSU/BLIT-circrna-pipeline, archived at Zenodo: https://doi.org/10.5281/zenodo.18920617) integrates cross-platform environment management, heterogeneous scripting languages (Bash, Python), and multi-step data analysis (quality control, alignment, circRNA identification, result aggregation). It systematically demonstrates BLIT’s capabilities in unified dependency management, safe execution of external commands, seamless bridging of multi-language components, and constructing clear, reliable pipeline logic. This reimplementation not only proves BLIT’s capability to handle real production-grade pipelines but also provides empirical evidence, through code comparison, of its substantial benefits in enhancing readability, maintainability, reproducibility, and lowering the barrier for R users to leverage command-line tools.

The stable operation log of this pipeline over tens of hours on a real dataset (RNA_phs003316, Nguyen *et al.* 2023) is provided as Table 5, available as supplementary data at *Bioinformatics Advances* online, further confirming its reliability and resource management capabilities in a production setting.

### 4.2 Custom tool wrapping and community-driven extension

BLIT supports extensibility through make_command(), which enables contributors to define new wrappers by instantiating customized R6 classes with execution logic, environment control, and parameter validation. Its modular, open-source design lowers barriers for collaboration: developers can propose or contribute tool wrappers via issue tracking, fostering an inclusive ecosystem that drives community engagement and sustainable tool evolution.

### 4.3 Native R object as command-line inputs

BLIT enables native R data structures to serve directly as inputs for command-line tools through its R6 class-based mechanism. Specifically, BLIT creates a dedicated R6 class wrapper for specific command-line tools like gistic2 (Mermel *et al.* 2011). When the user calls the wrapper function and passes in an R dataframe, the wrapper automatically handles data validation, serialization format conversion, temporary file management, and other underlying operations. For instance, when calling gistic2(my_dataframe, …):

1. BLIT first validates that my_dataframe is a proper data frame object.
2. It then serializes the data frame into the tab-separated format required by gistic2 and writes it to a temporary file.
3. It automatically constructs the gistic2 command-line arguments with the correct file paths.
4. Upon command completion, it automatically cleans up the temporary files.

This mechanism fundamentally eliminates the need for manual file conversion, preserves memory data integrity, and ensures seamless interoperability between R and shell toolchains.

## 5 Conclusion

BLIT significantly lowers barriers for biomedical researchers by unifying command-line workflows within R’s native environment. The package ensures cross-platform reproducibility through Micromamba and enables intuitive pipeline construction using R-native piping. By focusing on structured command encapsulation rather than high-level workflow scheduling, BLIT establishes a robust and transparent bridge between R analysis and external bioinformatics tools. This command-line integration strategy differentiates BLIT from alternative solutions, preserving the full power of native command-line tools while providing R’s usability advantages. It should be noted that checkpoint-resume functionality is currently under development in BLIT, and its community ecosystem is still in early stages—both areas represent key priorities for future development. We anticipate BLIT to serve as a foundational layer bridging R-based statistical workflows with command-line bioinformatics tools, enhancing accessibility for the broader life science community.

## Supplementary Material

vbag088_Supplementary_Data

## Data Availability

All code and data underlying this article are available in BLIT at https://github.com/WangLabCSU/blit/ and in BLIT-circrna-pipeline at https://github.com/WangLabCSU/BLIT-circrna-pipeline/. The pipeline can be accessed on Zenodo under the DOI: 10.5281/zenodo.18920617.

## References

[vbag088-B1] Csárdi G , Chang W. processx: Execute and Control System Processes. R Package Version 3.8.7. 2025. https://processx.r-lib.org. Accessed August 1, 2025.

[vbag088-B2] Di Tommaso P , Chatzou M, Floden EW et al Nextflow enables reproducible computational workflows. Nat Biotechnol 2017;35:316–9. 28398311 10.1038/nbt.3820

[vbag088-B3] Grüning B , Dale R, Sjödin A et al; Bioconda Team. Bioconda: sustainable and comprehensive software distribution for the life sciences. Nat Methods 2018;15:475–6. 29967506 10.1038/s41592-018-0046-7 PMC11070151

[vbag088-B4] Hester J , Bryan J. glue: Interpreted String Literals. R Package Version 1.8.0. 2024. https://CRAN.R-project.org/package=glue. Accessed August 1, 2025.

[vbag088-B5] Köster J , Rahmann S. Snakemake—a scalable bioinformatics workflow engine. Bioinformatics 2012;28:2520–2. 22908215 10.1093/bioinformatics/bts480

[vbag088-B6] Landau WM. The targets R package: a dynamic make-like function-oriented pipeline toolkit for reproducibility and high-performance computing. J Open Source Softw 2021;6:2959.

[vbag088-B7] Li H , Handsaker B, Wysoker A et al; 1000 Genome Project Data Processing Subgroup. The sequence alignment/map format and SAMtools. Bioinformatics 2009;25:2078–9. 19505943 10.1093/bioinformatics/btp352 PMC2723002

[vbag088-B8] Liu C , Ding S, Kim HJ et al Multitask benchmarking of single-cell multimodal omics integration methods. Nat Methods 2025;22:2449–60. 41083898 10.1038/s41592-025-02856-3 PMC12615258

[vbag088-B9] Mermel CH , Schumacher SE, Hill B et al GISTIC2.0 facilitates sensitive and confident localization of the targets of focal somatic copy-number alteration in human cancers. Genome Biol 2011;12:R41. 21527027 10.1186/gb-2011-12-4-r41 PMC3218867

[vbag088-B10] Nguyen VP , Campbell KM, Nowicki TS et al A pilot study of neoadjuvant nivolumab, ipilimumab and intralesional oncolytic virotherapy for HER2-negative breast cancer. Cancer Res Commun 2023;3:1628–37. 37621406 10.1158/2767-9764.CRC-23-0145 PMC10445661

[vbag088-B11] O’Sullivan DM , Doyle RM, Temisak S et al An inter-laboratory study to investigate the impact of the bioinformatics component on microbiome analysis using mock communities. Sci Rep 2021;11:10590. 34012005 10.1038/s41598-021-89881-2 PMC8134577

[vbag088-B12] Williams J , Pettorelli N, Dowell R et al SimpleMetaPipeline: breaking the bioinformatics bottleneck in metabarcoding. Methods Ecol Evol 2024;15:1949–57.

